# Cubature Information SMC-PHD for Multi-Target Tracking

**DOI:** 10.3390/s16050653

**Published:** 2016-05-09

**Authors:** Zhe Liu, Zulin Wang, Mai Xu

**Affiliations:** 1 School of Electronic and Information Engineering, Beihang University, Beijing 100191, China; liuzhe201@buaa.edu.cn (Z.L.); wzulin@buaa.edu.cn (Z.W.); 2 School of Information and Communication Engineering, North University of China, Taiyuan 030051, China; 3 Collaborative Innovation Center of Geospatial Technology, 129 Luoyu Road, Wuhan 430079, China

**Keywords:** Sequential monte carlo, probability hypothesis density, importance sampling, cubature information filter, Gaussian mixture, J0101

## Abstract

In multi-target tracking, the key problem lies in estimating the number and states of individual targets, in which the challenge is the time-varying multi-target numbers and states. Recently, several multi-target tracking approaches, based on the sequential Monte Carlo probability hypothesis density (SMC-PHD) filter, have been presented to solve such a problem. However, most of these approaches select the transition density as the importance sampling (IS) function, which is inefficient in a nonlinear scenario. To enhance the performance of the conventional SMC-PHD filter, we propose in this paper two approaches using the cubature information filter (CIF) for multi-target tracking. More specifically, we first apply the posterior intensity as the IS function. Then, we propose to utilize the CIF algorithm with a gating method to calculate the IS function, namely CISMC-PHD approach. Meanwhile, a fast implementation of the CISMC-PHD approach is proposed, which clusters the particles into several groups according to the Gaussian mixture components. With the constructed components, the IS function is approximated instead of particles. As a result, the computational complexity of the CISMC-PHD approach can be significantly reduced. The simulation results demonstrate the effectiveness of our approaches.

## 1. Introduction

### 1.1. Background

Multi-target tracking refers to sequential approximation of the states (positions, velocities, *etc.*), and the number of individual targets. It has been widely used in ground-moving-target tracking [1], visual tracking [2], and distribution fusion [3]. In multi-target tracking, both the state and observation sets of targets are time-varying. In practice, the associations between state and observation sets are always unknown, thus posing a challenge for multi-target tracking. The conventional approaches, such as the nearest-neighbour Kalman filter (NNKF) [4], Extended Kalman Filter (EKF) [5,6], multiple hypothesis tracking (MHT) [7], and joint probabilistic data association (JPDA) [8], are used to formulate the explicit associations between states and observations of targets. However, caused by the targets appearing and disappearing, the state and observation sets of the multi-target are uncertain. Such uncertainty costs high complexity in the conventional approaches on constructing the association between them. Several approaches have emerged to improve the performance of conventional approaches in terms of tracking accuracy [9,10,11,12,13].

Recently, filters based on random finite sets (RFS) have been developed as alternative frameworks of the traditional algorithms [14,15,16,17] to estimate the multi-target number and states. In RFS formulations, both the multi-target state and observation sets are modelled as random finite sets. Given such models, the multi-target tracking problem is formulated under the Bayesian framework [14]. Since the optimal RFS-based Bayesian filter is computationally intractable, the probability hypothesis density (PHD) filter has been proposed as a sub-optimal Bayesian filter for multi-target tracking [15,16,17]. By propagating the first order moment (namely the intensity), the PHD filter can save much computational complexity in comparison to the optimal RFS-based Bayesian filter. Moreover, it avoids the combinatorial problem arising from data association, which is the bottleneck for conventional multi-target tracking approaches to estimate multi-target number and states.

However, the difficulty of the PHD filter is that it is intractable to derive close-form solutions to the PHD filter formulations. To solve such difficulties, Vo *et al.* [18] proposed the bootstrap SMC-PHD (BSMC-PHD) filter, which selects the transition density as the IS function. The modified versions of BSMC-PHD filter have emerged in [19,20,21]. As for those BSMC-PHD filters, the IS function selection simplifies the weight computation in the prediction stage of the SMC-PHD filter. In practice, such a selection leads to a few particles with large-valued weights in the update stage, when targets have nonlinear motion [22].

To improve the tracking performance of the conventional SMC-PHD filter, several efficient approaches have been presented. Morelande *et al.* proposed the Rao-Blackwellised SMC-PHD (RB-SMC-PHD) filter in [23]. The RB-SMC-PHD filter utilizes several auxiliary variables to define the IS function, thus making it suitable for the intensity approximation of the SMC-PHD filter with conditionally linear Gaussian models. Motivated by the auxiliary particle filter, Whiteley *et al.* proposed the auxiliary particle PHD (APHD) filter in [24], in which the auxiliary variable is pre-selected to minimize the variance of the IS weights. Although the APHD filter enhances the efficiency of the SMC-PHD filter in nonlinear scenario, it yields poor performance in case of severe nonlinearities or high process noise [22]. Inspired by the unscented particle filter, Yoon *et al.* [25] utilized the unscented information filter (UIF) to design the IS function of SMC-PHD (called USMC-PHD filter). Since such a design takes the current observations of targets into account, it is more stable than the BSMC-PHD filter. However, the drawback of this filter is that its performance has been influenced by the selection of the sigma-points of UIF. Ristic *et al.* [20] proposed a novel state estimation method (called IBSMC-PHD), rather than partitioning particles in *ad-hoc* manner. Their method groups the particles in the update stage, thus enjoying the computational efficiency.

### 1.2. Our Work and Contributions

In this paper, we propose a cubature information SMC-PHD (CISMC-PHD) and its fast implementation (F-CISMC-PHD) approaches, which can be used to estimate the time-varying number and states of multi-target. As aforementioned, the disadvantage of conventional SMC-PHD filters is the tracking inefficiency in nonlinear scenario. To avoid such inefficiency, our CISMC-PHD approach applies the posterior intensity as the IS function. With such a selection, the current observations can be incorporated into the IS function design. Then, we utilize the cubature information filter (CIF) [26] with a gating method to calculate the IS function. Benefitting from tracking accuracy of CIF in high dimensional nonlinear case, the CISMC-PHD approach is capable of estimating the time-varying number and states of targets. To avoid initializing birth intensity in whole state space, a birth intensity initialization method is proposed for our CISMC-PHD approach. At last, we present the F-CISMC-PHD approach to reduce the computational complexity by considering groups of particles as Gaussian mixture components. These components are applied to approximate the IS functions instead of particles of the CISMC-PHD approach. Since the number of Gaussian components is much less than that of particles, the computational complexity can be greatly reduced. The main contributions of our work are listed as follows.

(1) We propose a novel IS function approximating method, which utilizes the CIF with a gating method to enhance the estimation accuracy of the SMC-PHD filter. Specifically, first, the posterior intensity is applied as the IS function of our CISMC-PHD approach, in order to incorporate the current observation set into the IS function approximation. Then, the gating method is integrated into the update step of the CIF for approximating the IS function. Benefitting from the most recent success of CIF in nonlinear state estimation, the tracking performance of the proposed CISMC-PHD approach is significantly enhanced.
(2) We develop a method to initialize the birth intensity for the next tracking recursion. Since the current estimated targets (*i.e.*, current survival targets) are not possible to be the birth targets at the next recursion, the observations of estimated targets are removed from the current observation set. Then, using an unbiased model, the remaining observations are mapped to state space for the birth intensity initialization. As such, the birth intensity can be adaptively initialized, making the target tracking more accurate and stable.
(3) We develop a fast version of the CISMC-PHD approach (namely F-CISMC-PHD). We first consider each group of particles as a Gaussian mixture component. Then these components are used to approximate the IS functions of the CISMC-PHD approach. With the approximated IS functions, the particles can be sampled from these components for the intensity prediction and update steps. As a result, the computational complexity of the proposed CISMC-PHD approach can be significantly reduced.

The rest of this paper is organized as follows. In Section 2, a brief overview of the SMC-PHD filter is provided. Section 3 proposes our CISMC-PHD approach. A fast implementation of CISMC-PHD approach is presented in Section 4. Simulation results are demonstrated in Section 5, and Section 6 concludes this paper.

## 2. A Brief Overview of The SMC-PHD Filter

In this section, we review the basic idea of the SMC-PHD filter in detail. The main notations used in this section are defined as follows.

$x_{k}$The state of a dynamic target at time *k*$z_{k}$The observation of a dynamic target at time *k*$\gamma_{k}\left(\cdot \right)$The intensity of birth target at time *k*$L_{k}$The number of the survival particles at time *k*$J_{k}$The number of the birth particles at time *k*$p_{s}\left(\cdot \right)$The survival probability of target$p_{d}\left(\cdot \right)$The detected probability of targetπ(·,·)The IS function of birth intensityq(·|·)The IS function of survival intensity

The SMC-PHD filter, motivated by the particle filter, is a sequential implementation of the PHD filter. In the SMC-PHD filter, the posterior intensity can be represented by a set of random samples of state vector $x_{k}$ with associated weights, which are usually called particles. By substituting these particles into the recursion of the PHD filter, the multi-dimensional integrals can be replaced by summations of the particles, which is computationally tractable.

More specifically, we define the particle set at time k-1 as ${\left\{x_{k-1}^{\left(i\right)},w_{k-1|k-1}^{\left(i\right)}\right\}}_{i=1}^{L_{k-1}}$, where $x_{k-1}^{\left(i\right)}$ and $w_{k-1|k-1}^{\left(i\right)}$ are the state and weight of the *i*-th particle at time k-1, respectively. The posterior intensity at time k-1 can be modelled by

(1) $$D_{k-1|k-1}\left(x_{k-1}|Z_{1:k-1}\right)=\sum_{i=1}^{L_{k-1}}w_{k-1|k-1}^{\left(i\right)}\cdot \delta \left(x-x_{k-1}^{\left(i\right)}\right)$$

where $Z_{1:k-1}$ is the multi-target observations from time 1 to k-1, and δ(·) is the Dirac Delta function. Notice that $Z_{k}=\left\{z_{k,1},z_{k,2},\ldots ,z_{k,M}\right\}$. Given $D_{k-1|k-1}\left(x_{k-1}|Z_{1:k-1}\right)$, the implementation of the SMC-PHD filter consists of the *prediction* and *update* stages.

**Prediction:** We first denote IS functions for the survival and birth targets as $q(x_{k}^{\left(i\right)}|x_{k-1}^{\left(i\right)},Z_{k})$ and $\pi (x_{k}^{\left(i\right)},Z_{k})$, respectively. Then, according to [18], the predicted intensity can be formulated by these IS functions,

(2) $$D_{k|k-1}\left(x_{k}|Z_{1:k-1}\right)=\sum_{i=1}^{L_{k-1}+J_{k}}w_{k|k-1}^{\left(i\right)}\cdot \delta \left(x-x_{k}^{\left(i\right)}\right)$$

where

(3) $$x_{k}^{\left(i\right)}∼\left\{\begin{array}{cc} q(x_{k}^{\left(i\right)}|x_{k-1}^{\left(i\right)},Z_{k}) & i=1,\ldots ,L_{k-1}\\ \pi (x_{k}^{\left(i\right)},Z_{k}) & i=L_{k-1}+1,\ldots ,L_{k-1}+J_{k} \end{array}\right.$$

(4) $$w_{k|k-1}^{\left(i\right)}\;=\;\left\{\begin{array}{cc} \frac{p_{s}\left(x_{k-1}^{\left(i\right)}\right)\cdot f\left(x_{k}^{\left(i\right)}|x_{k-1}^{\left(i\right)}\right)}{q(x_{k}^{\left(i\right)}|x_{k-1}^{\left(i\right)},Z_{k})}\; & \;i\;=\;1,2,\ldots ,L_{k-1}\\ \frac{\gamma (x_{k}^{\left(i\right)})}{J_{k}\cdot \pi \left(x_{k}^{\left(i\right)},Z_{k}\right)}\; & \;i\;=\;L_{k-1}\;+\;1,\ldots ,L_{k-1}\;+\;J_{k} \end{array}\right.$$

In Equation (4), $J_{k}$ is calculated by $J_{k}=\nu \int \gamma \left(x\right)dx$, where *ν* is the particle number of each birth target. Equations (2)–(4) can be then used to predict the states and weights of the particles.

**Update:** In this stage, we obtain the posterior intensity by updating Equation (2). Then, we have the following update strategy,

(5) $$D_{k|k}\left(x_{k}|Z_{1:k}\right)=\sum_{i=1}^{L_{k-1}+J_{k}}w_{k|k}^{\left(i\right)}\delta \left(x-x_{k}^{\left(i\right)}\right)$$

where

(6) $$w_{k|k}^{\left(i\right)}=\left(1-p_{d}\left(x_{k}^{\left(i\right)}\right)+\sum_{z\in Z_{k}}\frac{p_{d}\left(x_{k}^{\left(i\right)}\right)g_{k}\left(z|x_{k}^{\left(i\right)}\right)}{\kappa \left(z\right)+C_{z}}\right)w_{k|k-1}^{\left(i\right)}$$

κ(·) denotes the clutter intensity, and

(7) $$C_{z}=\sum_{i=1}^{L_{k-1}+J_{k}}p_{d}\left(x_{k}^{\left(i\right)}\right)g_{k}\left(z|x_{k,i}\right)w_{k|k-1}$$

Equations (2)–(6) include the main procedure of SMC-PHD at one recursion. Commonly, to avoid the degeneracy of particles, the resampling strategy is utilized to resample particle set ${\left\{x_{k}^{\left(i\right)},w_{k|k}^{\left(i\right)}\right\}}_{i=1}^{L_{k-1}+J_{k}}$. After resampling, we can use the clustering methods to extract number and states of targets.

## 3. The CISMC-PHD Approach for Multi-Target Tracking

In this section, we present our CISMC-PHD approach. To be more specific, we propose a novel IS function approximation algorithm incorporating the CIF and a gating method in Section 3.1. Then, Section 3.2 develops a method to initialize the birth intensity. Finally, Section 3.3 introduces the state extraction method for state estimation.

The nonlinear dynamic model of the target with state $x_{k}$ at time *k* is given as follows,

(8) $$\begin{array}{cc} \mathrm{Process}\mathrm{model}: & x_{k}=\phi \left(x_{k-1}\right)+v_{k-1} \end{array}$$

(9) $$\begin{array}{cc} \mathrm{Observation}\mathrm{model}: & z_{k}=\varphi \left(x_{k}\right)+w_{k} \end{array}$$

where ϕ(·) is the state transition function, and φ(·) denotes the relationship between state and observation. $v_{k-1}$ and $w_{k}$ are the process and observation noises at time k-1 and *k*, respectively. Both $v_{k-1}$ and $w_{k}$ are assumed to be Gaussian noises with zero means, and their covariances are denoted as $Q_{k-1}$ and $R_{k}$. According to this model, the transition density $f(x_{k}|x_{k-1})$ and likelihood $g_{k}\left(z_{k}|x_{k}\right)$ are subject to Gaussian distributions.

### 3.1. The IS Function Approximation Algorithm

As mentioned in Section 2, most of the conventional SMC-PHD filters utilize the transition density function as the IS functions, resulting in great tracking error for targets with nonlinear dynamics. A novel IS function approximation algorithm, incorporating the CIF with a gating method, is presented to improve the tracking accuracy.

In our approach, we select the IS functions of Equations (3) and (4) as

(10) $$\begin{array}{cc} \text{Survival IS}: & q\left(x_{k}^{\left(i\right)}|x_{k-1}^{\left(i\right)},Z_{k}\right)=N\left(x_{k}^{\left(i\right)};m_{k,s}^{\left(i\right)},P_{k,s}^{\left(i\right)}\right) \end{array}$$

(11) $$\begin{array}{cc} \text{Birth IS}: & \pi \left(x_{k}^{\left(i\right)}|Z_{k}\right)=N\left(x_{k}^{\left(i\right)};m_{k,b}^{\left(i\right)},P_{k,b}^{\left(i\right)}\right) \end{array}$$

where $m_{k,s}^{\left(\cdot \right)}$ and $m_{k,b}^{\left(\cdot \right)}$ are means of the survival and birth particles, respectively. $P_{k,s}^{\left(\cdot \right)}$ and $P_{k,b}^{\left(\cdot \right)}$ denotes the corresponding covariances of them.

Then, the problem of IS function design can be reduced to calculate $m_{k}^{\left(i\right)}$ and $P_{k}^{\left(i\right)}$. Now, we discuss on how to calculate them. For simplicity, they are replaced by $m_{k}$ and $P_{k}$, respectively. Here we use the CIF and gating methods to estimate them.

Before introducing the CIF method, we review the cubature rules [27]. The cubature rules are used to approximate the Gaussian weight integral. Assuming c(x) is a function on the n-dimension $R^{n}$, its Gaussian weight integral can be approximated by

(12) $$I_{N}\left(c\right)=\int_{R^{n}}c\left(x\right)N\left(x;m,P\right)\approx \frac{1}{2n}\sum_{j=1}^{2n}c\left(m+\sqrt{P}\alpha_{j}\right)$$

where

(13) $$\begin{array}{ccc} \alpha_{j} & = & \sqrt{n}{\left[1\right]}_{j} \end{array},j=1,2,\ldots ,2n$$

and ${\left[1\right]}_{j}$ is the *j*-th vector of the set

$$\left\{\left[\begin{array}{c} 1\\ 0\\ \vdots \\ 0 \end{array}\right],\cdots ,\left[\begin{array}{c} 0\\ 0\\ \vdots \\ 1 \end{array}\right],\left[\begin{array}{c} -1\\ 0\\ \vdots \\ 0 \end{array}\right],\left[\begin{array}{c} 0\\ 0\\ \vdots \\ -1 \end{array}\right]\right\}$$

According to Equation (12), the cubature rules can be used to compute the multi-dimension integrals in the prediction and update steps of the CIF method.

**Prediction:** In this step, we first predict the state $m_{k|k-1}$ and covariance $P_{k|k-1}$ according to cubature rules. Then, the predicted information state vector $y_{k|k-1}$ and matrix $Y_{k|k-1}$ are estimated for the *update* step.

Let $m_{k-1}$ and $P_{k-1}$ be the previous state and covariance, respectively. According to Equations (12) and (13), the *j*-th cubature point $\chi_{k-1,j}$ can be estimated by

(14) $$\chi_{k-1,j}=\sqrt{P_{k-1}}\alpha_{j}+m_{k-1}$$

Then, we can calculate $m_{k|k-1}$ and $P_{k|k-1}$ using the following formulations:

(15) $$\begin{array}{ccc} m_{k|k-1} & = & \frac{1}{2n}\sum_{j=1}^{2n}\chi_{k-1,j}^{*} \end{array}$$

(16) $$\begin{array}{ccc} P_{k|k-1} & = & \frac{1}{2n}\sum_{j=1}^{2n}\chi_{k-1,j}^{*}{\left(\chi_{k-1,j}^{*}\right)}^{T}-m_{k|k-1}{\left(m_{k|k-1}\right)}^{T}\\ & & +Q_{k-1} \end{array}$$

where ${\left(\cdot \right)}^{T}$ is the transpose operator, and

(17) $$\chi_{k-1,j}^{*}=\phi \left(\chi_{k-1,j}\right)$$

Given Equations (15) and (16), the information forms of $m_{k|k-1}$ and $P_{k|k-1}$ are represented [28] by

(18) $$y_{k|k-1}=Y_{k|k-1}m_{k|k-1}$$

and

(19) $$Y_{k|k-1}={\left(P_{k|k-1}\right)}^{-1}$$

where $y_{k|k-1}$ and $Y_{k|k-1}$ are the information state and matrix, respectively.

**Update:** We use the observation set $Z_{k}$ to update the predicted $y_{k|k-1}$ and $Y_{k|k-1}$ in the current step. In order to construct the associations between the observation set $Z_{k}$ and predicted observation $z_{k|k-1}$, a gating method is applied to extract the associated observations. With the extracted observations, we can finally obtain $m_{k}$ and covariance $P_{k}$.

We denote $z_{k|k-1}$ as the predicted observation, computed by

(20) $$\begin{array}{ccc} z_{k|k-1} & = & \frac{1}{2n}\sum_{j=1}^{2n}\chi_{k|k-1,j}^{*} \end{array}$$

where

(21) $$\chi_{k|k-1,j}^{*}=\varphi \left(\chi_{k|k-1,j}\right)$$

and

(22) $$\chi_{k|k-1,j}=\sqrt{P_{k|k-1}}\alpha_{j}+m_{k|k-1}$$

Utilizing the predicted observation $z_{k|k-1}$ of Equation (20), the error cross covariance matrix of state and observation can be evaluated by

(23) $$\begin{array}{ccc} P_{k|k-1}^{mz} & = & \frac{1}{2n}\sum_{j=1}^{2n}\left(\chi_{k|k-1,j}-m_{k|k-1}\right){\left(\chi_{k|k-1,j}^{*}-z_{k|k-1}\right)}^{T}\\ & = & \frac{1}{2n}\sum_{j=1}^{2n}\chi_{k|k-1,j}{\left(\chi_{k|k-1,j}^{*}\right)}^{T}-m_{k|k-1}{\left(z_{k|k-1}\right)}^{T} \end{array}$$

With the above obtained parameters, we can calculate the state contribution and its corresponding information matrix as

(24) $$i_{k,j}=Y_{k|k-1}P_{k|k-1}^{mz}R_{k}^{-1}\left(\mu_{j}+{\left(Y_{k|k-1}P_{k|k-1}^{mz}\right)}^{T}m_{k|k-1}\right)$$

and

(25) $$I_{k}=Y_{k|k-1}P_{k|k-1}^{mz}R_{k}^{-1}{\left(Y_{k|k-1}P_{k|k-1}^{mz}\right)}^{T}$$

where $\mu_{j}$ is the innovation of the *j*-th observation $z_{j}$ ($z_{j}\in Z_{k}$), expressed by

(26) $$\mu_{j}=z_{j}-z_{k|k-1}$$

In our scenario, $z_{j}$ is a two-dimension vector, and $\mu_{j}$ follows a two-dimension Gaussian distribution.

In practice, the observation set may contain large clutters. The existence of these clutters cannot only degenerate the estimation accuracy, but also increase the computational complexity. Recently, several gating technologies have been proposed to remove the clutters from the observation set [29,30]. Inspired by [30], we utilize the gating technology to reduce the influence of clutters.

Intuitively, observations far away from the predicted observation are subject to be generated by clutters. These observations must be removed from the observation set. With the gating technology, the left observations can be represented by

(27) $$\begin{array}{cc} {\hat{Z}}_{k}\;=\;\left\{z_{k,j}|\mu_{j}^{T}{\left(P_{k|k-1}^{zz}\right)}^{-1}\mu_{j}<\sqrt{T_{h}}\right\} & z_{k,j}\in Z_{k} \end{array}$$

where $P_{k|k-1}^{zz}$ is the covariance matrix of the predicted observation $z_{k|k-1}$, and ${\left(\cdot \right)}^{-1}$ is the matrix inversion. $T_{h}$ is the threshold of the gate. According to Equation (27), the innovation $\mu_{j}$ follows the Chi-square distribution. Thus, $T_{h}$ can be determined by the dimension of $\mu_{j}$ and association probability. Commonly, the square root of $T_{h}$ is known as the number of Sigma. Literature [30] proved that the number of Sigma gates ranging from 3 to 5 (corresponding to $T_{h}=9-25$) can guarantee the true observation lying inside the gate with “enough” probability (≥0.971), when the dimension of $\mu_{j}$ is less than three. In this paper, we select the number of Sigma gates being to 4 (corresponding to $T_{h}=16$). When the dimension of $\mu_{j}$ is less than three, such a selection guarantee that the association probability ≥0.998.

Then, we concentrate on computing $P_{k|k-1}^{zz}$ of Equation (27). Let $P_{k|k-1}^{mz}$ be the cross covariance matrix between observation and state space. According to the linear error propagating of [31], $P_{k|k-1}^{mz}$ can be rewritten as

(28) $$P_{k|k-1}^{mz}≃P_{k|k-1}H_{k}^{T}$$

where $H_{k}$ is the linearized matrix.

Obviously, $H_{k}$ can be approximated by

(29) $$H_{k}≃{\left(P_{k|k-1}^{mz}\right)}^{T}P_{k|k-1}^{-1}$$

With the achieved $H_{k}$, according to [32], $P_{k|k-1}^{zz}$ can be calculated by

(30) $$P_{k|k-1}^{zz}=H_{k}P_{k|k-1}H_{k}^{T}+R_{k}$$

Substituting the achieve $P_{k|k-1}^{zz}$ into Equation (27), ${\hat{Z}}_{k}$ can be extracted from the current observation set $Z_{k}$.

With the extracted observation set ${\hat{Z}}_{k}$, the information state vector $y_{k}$ and matrix $Y_{k}$ are represented as:

(31) $$\begin{array}{c} y_{k}=y_{k|k-1}+\sum_{j=1}^{|{\hat{Z}}_{k}|}i_{k,j} \end{array}$$

(32) $$\begin{array}{c} Y_{k}=Y_{k|k-1}+\sum_{j=1}^{|{\hat{Z}}_{k}|}I_{k,j} \end{array}$$

Given information state $y_{k}$ and matrix $Y_{k}$, posterior state $m_{k}$ can be reconstructed based on Equation (18) :

(33) $$m_{k}={Y_{k}}^{-1}y_{k}$$

Moreover, the posterior covariance $P_{k}$ is recovered based on Equation (19):

(34) $$P_{k}={\left(Y_{k}\right)}^{-1}$$

If there is no observation that lies inside the gate (${\hat{Z}}_{k}=\emptyset$), we approximate $m_{k}$ and $P_{k}$ in the following,

(35) $$\begin{array}{c} m_{k}=m_{k|k-1} \end{array}$$

(36) $$\begin{array}{c} P_{k}=P_{k|k-1} \end{array}$$

Substituting the above obtained $m_{k}$ and $P_{k}$ into Equations (10) and (11), we can approximate the IS functions of survival and birth targets for our CISMC-PHD approach.

### 3.2. The Birth Intensity Initialization Method

According to Equation (2), the birth intensity has large effect on the posterior intensity estimation. Targets may “born at anywhere” of the state space. In other words, birth intensity γ(x) may cover the whole state space, which is rather exhaustive [25]. To avoid such a disadvantage, observation-driven birth intensity initiation methods were proposed [20,33,34]. Inspired by these methods, an adaptive birth intensity initialization method is proposed for the CISMC-PHD approach. Instead of initializing birth intensity across the whole state space, the proposed method of CISMC-PHD approach utilizes the current observations and estimated targets to initialize the birth intensity at the next recursion. Compared with the conventional SMC-PHD filters, our method can initialize the birth intensity without knowing it as a prior.

The implementation of our method consists of two steps. First, in order to initialize the birth intensity, we remove observations generated by the estimated targets That is because the current survival targets cannot be new-born targets at the next recursion. Second, we use the remaining observations to estimate the birth target components, which can be used to calculate the birth intensity. With these two steps, the birth intensity can be initialized for the next recursion.

**Step1. Remove observations generated by the estimated targets.**

In the basic PHD filter, it is assumed that each target can yield at most one observation [35]. According to this assumption, each target has one and only one corresponding observation. Influenced by the noises and clutters, the observation generated by the target may appear around the target. In other words, observations around the target has the large probability to be generated by the same target. Therefore, the birth target state set can be estimated by removing states of estimated targets from the multi-target state.

Here, we adopt the bearing and range tracking model [36] to illustrate the birth intensity initialization method of our CISMC-PHD approach. Let $x_{k,i}^{e}$ be the state of the *i*-th target in the estimate state set $X_{k}^{e}$. $x_{k,i}^{e}$ consists of position and velocity, while $z_{k,j}$ ($z_{k,j}\in Z_{k}$) consists of the bearing angle and range. We define the distance between $x_{k,i}^{e}$ and $z_{k,j}$ ($z_{k,j}\in Z_{k}$) as

(37) $$d_{i,j}=\left|{\left(z_{k,j}-\varphi \left(x_{k,i}^{e}\right)\right)}_{r}\right|$$

where ${\left(\cdot \right)}_{r}$ denotes the range-dimension element, and |·| is the absolution value.

We follow the way of [30] to select the certain threshold for Equation (37),

(38) $$d_{i,j}<l\cdot \sigma_{r}$$

where $\sigma_{r}$ is the error of the range-dimension (known as a prior). *l* is the confidence level, commonly selected from l=3∼5. Here, we use l=3, which can guarantee that the associated probability equals to 0.997.

With Equations (37) and (38), we can remove the observations associated with the estimated targets. Let ${\tilde{Z}}_{k}$ be the observations of birth targets, the removing procedure is summarized in Table 1.

**Step2. Estimate the birth target components.**

Once ${\tilde{Z}}_{k}$ is obtained, we turn to estimate the birth target components (the mean of the *i*-th target state vector $m_{k,b}^{\left(i\right)}$ and its corresponding covariance $P_{k,b}^{\left(i\right)}$) by the unbiased model of [37].

Let ${\tilde{z}}_{k,i}\in {\tilde{Z}}_{k}$, we map ${\tilde{z}}_{k,i}$ into state space denoted by ${\tilde{z}}_{k,i}^{c}={\left[p_{k,i}^{x},p_{k,i}^{y}\right]}^{T}$. $p_{k,i}^{x}$ and $p_{k,i}^{y}$ can be computed by $p_{k,i}^{x}=\beta_{\theta }^{-1}r_{k,i}\cos\theta_{k,i}$ and $p_{k,i}^{y}=\beta_{\theta }^{-1}r_{k,i}\sin\theta_{k,i}$. $\beta_{\theta }=\sigma_{\theta }$ is a biased comparison factor, where $\sigma_{\theta }$, as a prior, is the error of bearing angle $\theta_{k,i}$. According to [37], $m_{k}^{\left(i\right)}$, the mean of the *i*-th birth target state, can be estimated as

(39) $$m_{k}^{\left(i\right)}={\left[p_{k,i}^{x},0,p_{k,i}^{y},0,0\right]}^{T}$$

The covariance can be approximated by

(40) $$P_{k}^{\left(i\right)}=\left[\begin{array}{ccccc} \sigma_{xx} & 0 & \sigma_{xy} & 0 & 0\\ 0 & \sigma_{v} & 0 & 0 & 0\\ \sigma_{yy} & 0 & \sigma_{xy} & 0 & 0\\ 0 & 0 & 0 & \sigma_{v}^{2} & 0\\ 0 & 0 & 0 & 0 & \sigma_{\theta }^{2} \end{array}\right]$$

where $\sigma_{v}$, as a prior, is the standard deviation of velocity. In Equation (40), the following exists,

(41) $$\left\{\begin{array}{cc} \sigma_{xx}= & \left(\beta_{\theta }^{-2}-2\right){\left({\tilde{r}}_{k,i}\right)}^{2}\cos^{2}\left(\theta_{k,i}\right)+0.5({\left({\tilde{r}}_{k,i}\right)}^{2}\\ & +\sigma_{r}^{2})\left(1+\beta_{\theta }^{4}\cos\left(2\theta_{k,i}\right)\right)\\ \sigma_{xy}= & \left(\beta_{\theta }^{-2}-2\right){\left({\tilde{r}}_{k,i}\right)}^{2}\cos\left(\theta_{k,i}\right)\sin\left(\theta_{k,i}\right)+0.5({\left({\tilde{r}}_{k,i}\right)}^{2}\\ & +\sigma_{r}^{2})\left(1+\beta_{\theta }^{4}\cos\left(2\theta_{k,i}\right)\right)\\ \sigma_{yy}= & \left(\beta_{\theta }^{-2}-2\right){\left({\tilde{r}}_{k,i}\right)}^{2}\sin^{2}\left(\theta_{k,i}\right)+0.5({\left({\tilde{r}}_{k,i}\right)}^{2}\\ & +\sigma_{r}^{2})\left(1-\beta_{\theta }^{4}\cos\left(2\theta_{k,i}\right)\right) \end{array}\right.$$

Finally, we can construct the new-born targets as ${\left\{m_{k,i},P_{k,i}\right\}}_{i=1}^{N_{k},b}$, where $N_{k,b}=\left|{\tilde{Z}}_{k}^{c}\right|$ is considered as the number of birth targets. We use Equation (11) to sample states of birth particles. The weights of these birth particles are initialized with the same values, $w_{k,b}^{\left(i\right)}=\frac{\int \gamma (x)dx}{N\cdot N_{k,b}}$, where *N* is the number particles for each target, and γ(x) is defined in Section 2. On this basis, these birth particles become survival particles at time k+1. That is to say, the IS functions of these particles at time k+1 can be computed by the method of Section 3.1. Notice that the new-born target in this section may contain clutters, and these clutters can be removed in the resampling step of the CISMC-PHD approach.

The proposed initialization method may cause overestimation of targets. To overcome the issue of overestimation, some advanced methods, such as [33,34], *etc.*, may be incorporated for initialization of our approach. It is an interesting future work.

### 3.3. State Estimation

In multi-target tracking, it is rather important to estimate the target number and states. As for the state estimation, clustering methods, are commonly used in SMC-PHD filters [16,18]. However, they are subject to biased estimation [21]. Ristic *et al.* [21] proposed an method that clusters the particles into several groups at the *update* stage. In this paper, we intend to adopt the method of [38] for state estimation, which is an improved method of [21]. There are also several alternative methods, such as Zhao’s method [39] and MEAP method [40], which have better estimation performance.

According to Equation (6), the updated weight $w_{k}^{i}$ of the *i*-th particle consists of two parts,

(42) $$w_{k}^{\left(i,j\right)}=\left\{\begin{array}{cc} \left(1-p_{d}\left(x_{k}\right)\right)w_{k|k-1}^{\left(i\right)}\; & \;j=0\\ \frac{p_{d}\left(x_{k}^{\left(i\right)}\right)g_{k}\left(z_{k,j}|x_{k}^{\left(i\right)}\right)}{\kappa \left(z_{k,j}\right)+C_{z_{k,j}}}w_{k|k-1}^{i}\; & \;j=1,\ldots ,|{\hat{Z}}_{k}| \end{array}\right.$$

In Equation (42), j=0 denotes that there is no observation, and $C_{z_{k,j}}$ can be computed by Equation (7). For state estimation, we aggregate $W^{j}$ of particle weights corresponding to observation $z_{k,j}$,

(43) $$\begin{array}{cc} W^{j}=\sum_{i=1}^{L_{k-1}+J_{k}}w_{k}^{\left(i,j\right)} & j=0,\ldots ,|{\hat{Z}}_{k}| \end{array}$$

According to Equations (42) and (43), if $z_{k,j}$ is generated by the clutter, then the likelihood $g_{k}\left(z_{k,j}|x_{k}^{\left(i\right)}\right)$ may be small, leading to low value of $W^{j}$. However, if $z_{k,j}$ is generated by the target, then $W^{j}$ may be large due to the large value of $g_{k}\left(z_{k,j}|x_{k}^{\left(i\right)}\right)$. Thus, setting certain threshold $W_{th}$ for $W^{j}$, we can assign particles $\left\{x_{k,j}^{\left(i\right)},w_{k}^{\left(i,j\right)}\right\}$ that satisfy $W^{j}>W_{th}$ to the *j*-th target. In this paper, we set $W_{th}=0.5$, the same as [21]. Then, $x_{k,j}$ and $P_{k,j}$ can be calculated in the following

(44) $$\begin{array}{ccc} x_{k,j} & = & \frac{1}{W^{j}}\sum_{i=1}^{L_{k-1}+J_{k}}w_{k}^{i,j}x_{k,j}^{\left(i\right)} \end{array}$$

(45) $$\begin{array}{ccc} P_{k,j} & = & \frac{1}{W^{j}}\sum_{i=1}^{L_{k-1}+J_{k}}w_{k}^{i,j}\left(x_{k,j}^{\left(i\right)}-x_{k,j}\right)\left(x_{k,j}^{\left(i\right)}-x_{k,j}\right) \end{array}$$

Given Equations (44) and (45), the states of multi-target can be finally output. We summarize our approach at time *k* in Table 2.

## 4. A Fast Approach For The CISMC-PHD Filter

**Figure 1:**
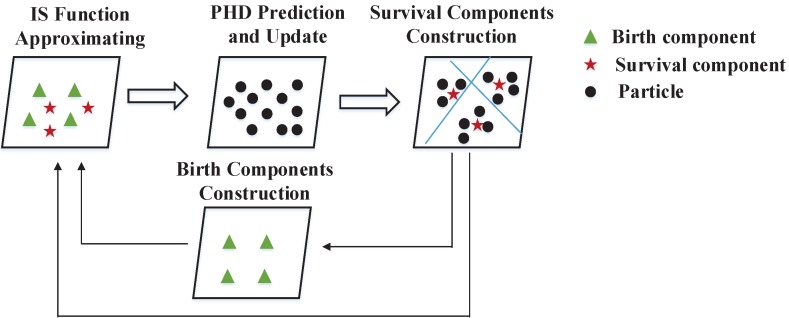
Framework of the F-CISMC-PHD approach. In the *IS function Approximating* step, we utilize the survival and birth components to approximate the IS functions. Then the predicted particles are generated and updated in the *PHD prediction and update* step to achieve the posterior particles. By clustering the particles into several groups, the Gaussian Mixture components (namely survival components) can be constructed in the *survival components construction* step. Meanwhile, we also apply the survival components to estimate the birth components in the *birth components construction* step. These components are used to approximate the IS functions in the next iteration.

In this section, we focus on reducing the computational complexity of our CISMC-PHD approach. Section 4.1 presents a fast implementation of the CISMC-PHD approach, namely the F-CISMC-PHD approach. Then, we analyze the computational complexity of the CISMC-PHD and F-CISMC-PHD approaches in Section 4.2. The framework of the improved approach is illustrated in Figure 1.

### 4.1. The Fast CISMC-PHD Filter

In this section, we introduce the F-CISMC-PHD approach, which can save the computational time of the CISMC-PHD approach. Inspired by [41], we consider the particle groups of targets as the Gaussian mixture components. Recall that $N_{k-1,b}$ is the number of birth components. $m_{k-1,b}^{\left(i\right)}$ and $P_{k-1,b}^{\left(i\right)}$ denote the mean and covariance of the *i*-th birth component, respectively. The posterior intensity of Equation (1) can be approximated by

(46) $$D_{k-1|k-1}\left(x_{k-1}|Z_{1:k-1}\right)\approx \sum_{i=1}^{N_{k-1,s}}G_{k-1,s}^{\left(i\right)}N\left(x_{k-1};m_{k-1,b}^{\left(i\right)},P_{k-1,b}^{\left(i\right)}\right)+\sum_{i=1}^{N_{k-1,b}}G_{k-1,b}^{\left(i\right)}N\left(x_{k-1};m_{k-1}^{\left(i\right)},P_{k-1}^{\left(i\right)}\right)$$

where $m_{k-1,s}^{\left(i\right)}$ and $P_{k-1,s}^{\left(i\right)}$ are the mean and covariance of the *i*-th survival component, respectively. $G_{k-1,s}^{\left(i\right)}$ is its corresponding weight, $N_{k-1,s}$ is the number of survival components, and $G_{k-1,b}^{\left(i\right)}$ is the weight of the *i*-th birth component. Here, $G_{k-1,b}^{\left(i\right)}=\frac{\int \gamma (x)dx}{N_{k-1,b}}$.

Commonly, birth components at time k-1 become survival components at time *k*. We combine birth and survival components into one set. That is ${\left\{G_{k-1}^{\left(i\right)},m_{k-1}^{\left(i\right)},P_{k-1}^{\left(i\right)}\right\}}_{i=1}^{N_{k-1,b}+N_{k-1,s}}={\left\{G_{k-1,b}^{\left(i\right)},m_{k-1,b}^{\left(i\right)},P_{k-1,b}^{\left(i\right)}\right\}}_{i=1}^{N_{k-1,b}}\cup {\left\{G_{k-1,s}^{\left(i\right)},m_{k-1,s}^{\left(i\right)},P_{k-1,s}^{\left(i\right)}\right\}}_{i=1}^{N_{k-1,s}}$, where $G_{k-1}^{\left(i\right)},m_{k-1}^{\left(i\right)},P_{k-1}^{\left(i\right)}$ denote the weight, mean and covariance of the *i*-th combined component.

With the combined components, we use the CIF method of Section 3.1 to approximate the IS function of each component. Here, we utilize $q(x|m_{k-1}^{\left(i\right)},Z_{k})$ to represent the IS function of the *i*-th component. On this basis, the *j*-th predicted particle, which is generated by the *i*-th component, can be represented by

(47) $$x_{k}^{\left(j\right)}∼\begin{array}{cc} q(x_{k}^{\left(j\right)}|m_{k-1}^{\left(i\right)},Z_{k}) & j=\sum_{a=1}^{i-1}\left\lfloor G_{k-1}^{\left(a\right)}\cdot N\right\rfloor +1,\sum_{a=1}^{i-1}\left\lfloor G_{k-1}^{\left(a\right)}\cdot N\right\rfloor +2,\ldots ,\sum_{a=1}^{i}\left\lfloor G_{k-1}^{\left(a\right)}\cdot N\right\rfloor \end{array}$$

(48) $$w_{k|k-1}^{\left(j\right)}\;=\;\begin{array}{cc} \frac{p_{s}\left(x_{k-1}^{\left(i\right)}\right)\cdot f\left(x_{k}^{\left(j\right)}|x_{k-1}^{\left(i\right)}\right)}{q(x_{k}^{\left(j\right)}|m_{k-1}^{\left(j\right)},Z_{k})}\cdot \frac{G_{k-1}^{\left(a\right)}}{\left\lfloor G_{k-1}^{\left(a\right)}N\right\rfloor }\; & \;j=\sum_{a=1}^{i-1}\left\lfloor G_{k-1}^{\left(a\right)}\cdot N\right\rfloor +1,\sum_{a=1}^{i-1}\left\lfloor G_{k-1}^{\left(a\right)}\cdot N\right\rfloor +2,\ldots ,\sum_{a=1}^{i}\left\lfloor G_{k-1}^{\left(a\right)}\cdot N\right\rfloor \end{array}$$

where $f\left(x_{k}^{\left(j\right)}|x_{k-1}^{\left(i\right)}\right)=N\left(x_{k}^{\left(j\right)};m_{k|k-1}^{\left(i\right)},P_{k|k-1}^{\left(i\right)}\right)$. In addition, $m_{k|k-1}^{\left(i\right)}$ and $P_{k|k-1}^{\left(i\right)}$ are the predicted mean and covariance of *i*-th component, respectively, which can be computed by Equations (15) and (16). ⌊·⌋ denotes the nearest floor integer, and $\left\lfloor G_{k-1}^{\left(i\right)}\cdot N\right\rfloor$ is the number of particles generated by the *i*-th component. According to Equations (47) and (48), the number of predicted particles is $N_{k}=\sum_{a=1}^{N_{k,s}+N_{k,b}}\left\lfloor G_{k-1}^{\left(a\right)}\cdot N\right\rfloor$.

Then, the predicted weight of Equation (48) is substituted into Equation (42) for particle grouping and state estimation. Recall that, weight $W^{j}$ of Equation (43) can be used to assign the particles into the *j*-th group, where the mean $x_{k,j}$ and covariance $P_{k,j}$ can be calculated by Equations (44) and (45) in Section 3.3. Given $W^{j}$, $x_{k,j}$ and $P_{k,j}$, we model the *j*-th group as a Gaussian mixture component $\left\{G_{k}^{\left(j\right)},x_{k,j},P_{k,j}\right\}$, where we set $G_{k}^{\left(j\right)}=\frac{W_{j}}{\max_{j}W_{j}}$. With such a selection, it can guarantee that the groups with large $W^{j}$ have enough number of sampling particles. Note that the group with small $W^{j}$ has the large probability to be generated by the clutter, and such a group should be neglected to avoid the waste of computational time. We set a certain threshold $T_{g}$ for $\left\{G_{k}^{\left(j\right)}|G_{k}^{\left(j\right)}>T_{g}\right\}$, subject to $T_{g}\cdot N\ll N$. In this paper, we set $T_{g}=0.1$. The construction procedure of the target components is summarized in Table 3.

The target components of Table 3 refer to the survival target components. The birth components and target state estimation can be achieved by Section 3.2 and Section 3.3, respectively.

### 4.2. Computational Complexity

In this section, we analyze the computational complexity of the CISMC-PHD, F-CISMC-PHD and conventional SMC-PHD approaches. For justice, we adopt the same state estimation and birth target initialization methods for the three approaches. The computational complexity of the three approaches on state estimating and birth target initializing is the same, when they have same particle numbers and observations. Thus, it can be neglected for the computational complexity comparing. In addition, we select the multinomial resampling method as the resampling methods of the CISMC-PHD and conventional SMC-PHD approaches. The particle numbers of these approaches are equal to $N_{p}$.

We begin with the computational complexity analysis of the CISMC-PHD approach. Incorporating the CIF and gating methods into the SMC-PHD approach, the CISMC-PHD approach can achieve a good estimation accuracy in nonlinear target tracking. As mentioned in Section 3.1, each particle is applied to approximate the IS functions. The computational complexity of the CIF and gating method per particle is nearly $O(n_{d}^{3})$, where $n_{d}$ is the dimension of the particle state. The computational complexity of the CIF-based IS functions is $O(N_{p}\cdot n_{d}^{3})$. Besides, the computational complexity of the PHD update step is $O(N_{p}\cdot M)$, where *M* is the number of observations. In addition, the resampling step of our CISMC-PHD approach consumes $O(N_{p})$ computational complexity. Thus, the computational complexity of our CISMC-PHD approach in total is $O(N_{p}\cdot n_{d}^{3}+N_{p}\cdot M+N_{p})$.

Then, we turn to the analysis of the computational complexity of the F-CISMC-PHD approach. Since the F-CISMC-PHD approach adopts the target components to compute the CIF-based IS functions, the computational complexity of the CIF-based IS functions is $O(N_{G}\cdot n_{d}^{3})$, where $N_{G}$ is the number of target components, $N_{G}\ll N_{p}$. Assuming that in the PHD prediction step, $N_{G}$ target components generate $N_{p}$ particles. The computational complexity of the PHD update step is $O(N_{p}\cdot M)$. Besides, the Gaussian target component forming consumes O(M+1). Combining the computational complexity of these steps together, the computational complexity of the F-CISMC-PHD approach is $O(N_{G}\cdot n_{d}^{3}+N_{p}\cdot M+M+1)$. In practice, $M\ll N_{p}$, the computational complexity of F-CISMC-PHD filter is much less than the CISMC-PHD filter.

The conventional SMC-PHD approach uses the transitional density as the IS function. Compared with the CISMC-PHD and F-CISMC-PHD approaches, it does not need to the IS function computing. Hence, the computational complexity of the conventional SMC-PHD approach can be approximated as $O(N_{p}\cdot M+N_{p})$, where $O(N_{p}\cdot M)$ and $O(N_{p})$ are the computational complexity of the update and resampling steps. Obviously, $O\left(N_{p}\cdot M+N_{p}\right)<O\left(N_{G}\cdot n_{d}^{3}+N_{p}\cdot M+M+1\right)<O\left(N_{p}\cdot n_{d}^{3}+N_{p}\cdot M+N_{p}\right)$. Thus, the computational complexity of the conventional SMC-PHD approach is smaller than the CISMC-PHD and F-CISMC-PHD approaches.

With the above discussion, we can conclude that the conventional SMC-PHD approach has the lowest computational complexity, and the CISMC-PHD approach has the highest computational complexity. However, the estimation accuracy of the conventional SMC-PHD approach is the lowest, and the estimation accuracy of the CISMC-PHD approach is the highest. The F-CISMC-PHD approach can make a trade-off between the computational complexity and estimation accuracy. Such a conclusion can be observed in Section 5.

## 5. Simulation Results

In this section, we validate the tracking performance of the proposed CISMC-PHD and F-CISMC-PHD approaches. In Section 5.1, a nonlinear simulation scenario composed of five targets is constructed. Then, Section 5.2 compares the estimation results of the IBSMC-PHD [20], proposed CISMC-PHD and F-CISMC-PHD approaches, in terms of the optimal subpattern assignment (OSPA) metric [42] and Root Mean Square Error (RMSE). At last, we compare the simulation results of all three approaches with different numbers of clutters and detection probabilities, to validate the effectiveness of our approaches.

### 5.1. Simulation Scenarios

In our simulations, we use the nonlinear scenario, the same as [36]. Let x be the target states, represented by $x={\left[p_{x},v_{x},p_{y},v_{y},\alpha \right]}^{T}$. In this paper, $\left(p_{x},p_{y}\right)$ is the position, $\left(v_{x},v_{y}\right)$ is the velocity, and *α* is the turn rate. With the above definitions, we model the nonlinear dynamic equation as

(49) $$\begin{array}{cc} x_{k}= & \\ & \left[\begin{array}{ccccc} 1 & \frac{\sin(\alpha_{k-1}T)}{\alpha_{k-1}} & 0 & -\frac{1-\cos(\alpha_{k-1}T)}{\alpha_{k-1}} & 0\\ 0 & \cos(\alpha_{k-1}T) & 0 & -\sin(\alpha_{k-1}T) & 0\\ 0 & \frac{1-\cos(\alpha_{k-1}T)}{\alpha_{k-1}} & 1 & \frac{\sin(\alpha_{k-1}T)}{\alpha_{k-1}} & 0\\ 0 & \sin(\alpha_{k-1}T) & 0 & \cos(\alpha_{k-1}T) & 0\\ 0 & 0 & 0 & 0 & 1 \end{array}\right]x_{k-1}\\ & +\left[\begin{array}{ccc} \frac{T^{2}}{2} & 0 & 0\\ T & 0 & 0\\ 0 & \frac{T^{2}}{2} & 0\\ 0 & T & 0\\ 0 & 0 & 1 \end{array}\right]\varepsilon_{k-1} \end{array}$$

where T=1 second (s) is the sampling interval. In addition, $\varepsilon_{k-1}$ is the noise, defined by $\varepsilon_{k-1}∼N\left(\varepsilon_{k-1};0,Q\right)$. We denote $Q=diag(\sigma_{x,\varepsilon }^{2},\sigma_{y,\varepsilon }^{2},\sigma_{\alpha }^{2})$ as covariance matrices of $\varepsilon_{k-1}$. In this paper, we set $\sigma_{x,\varepsilon }=\sigma_{y,\varepsilon }=1$ meter/second${}^{2}$ (m/s${}^{2}$), $\sigma_{\alpha }=\pi /180$ rad. The initial states, appearing times, and disappearing times of targets are listed in Table 4.

**Figure 2:**
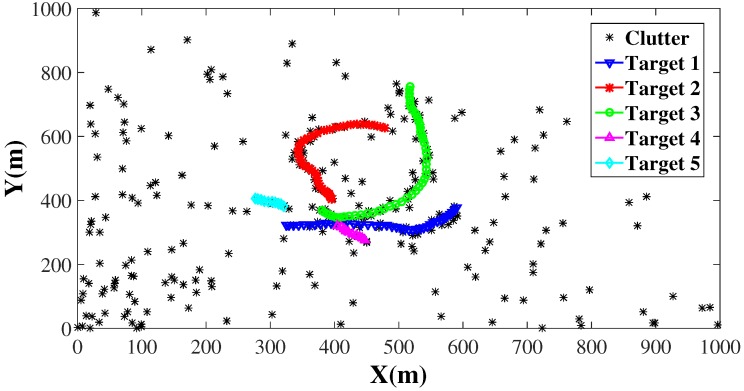
Ground-truth trajectories of five targets with the clutter number setting to be 10. The target trajectories are depicted by circle-solid lines with different colors, while the asterisks denote clutters.

Besides, the observation model is given by

(50) $$z_{k}=\left[\begin{array}{c} \arctan\left(\frac{p_{y}}{p_{x}}\right)\\ \sqrt{p_{x}^{2}+p_{y}^{2}} \end{array}\right]+\eta_{k}$$

where $\eta_{k}$ is the observation noise defined by $\eta_{k}∼N\left(\eta_{k};0,R\right)$, and $R=diag(\sigma_{\theta }^{2},\sigma_{r}^{2})$ is the covariance. Here, we set $\sigma_{\theta }=\pi /180$ rad and $\sigma_{r}=1$ meter (m). We also assume that clutters are uniformly distributed in the detection region, where the angle range is (0,π/2) rad, and distance range is (0,1000) m. Trajectories of the five targets in both scenarios are shown in Figure 2, where the clutter number is set to be 10 for each scenario.

For parameters, we set the gating threshold $T_{h}=16$ according to [32], the probability of detection and survival are $p_{d}\left(x_{k}\right)=0.95$, and $p_{s}\left(x_{k}\right)=0.99$, in accordance with [36]. The particle numbers for each birth target and survival targets are 5 and 100, respectively. All of the simulations are run in a computer with MATLAB 2015a, and i5 3.2 GHz processor with 4GB RAM.

### 5.2. Comparison of Estimation Accuracy on Certain Number of Clutters

To compare the estimation accuracy, we adopt the first order OSPA and RMSE as the metric. Here, we discuss the first order OSPA distance in the following. Let $X=\{x_{1},\ldots ,x_{n}\}$ and $Y=\{y_{1},\ldots ,y_{n}\}$ be two RFSs, where *m* and *n* are numbers of elements in X and Y, respectively. Supposing that $\Omega_{n}$ represents the set of permutations of {1,2,…,n}, the first order OSPA metric can be rewritten by

(51) $${\bar{d}}_{p}^{c}\left(X,Y\right)=\frac{1}{n}\left(\min_{\varsigma \in \Omega_{n}}\sum_{i=1}^{m}d^{c}{\left(x_{i},y_{\varsigma (i)}\right)}^{p}+c^{p}\left(n-m\right)\right)$$

where $d^{c}\left(x,y\right)=\min\left(c,d\left(x,y\right)\right)$, c>0 is a cut-off factor, and d(x,y) is the distance between x and y. In this paper, we set c=150 in accordance with [43], and use the Euclidean distance to compute d(x,y).

**Figure 3:**
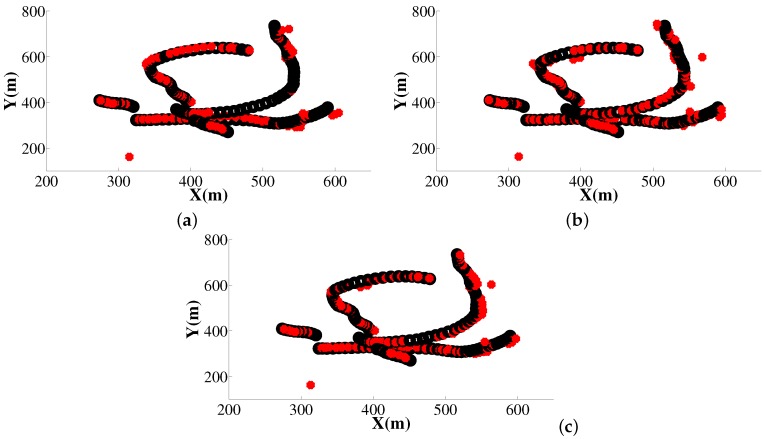
Estimated trajectories of three approaches with clutter number being 10. The estimated trajectories are represented with the red (light) points, while the true trajectories are with the black (dark) solid line. (**a**) Trajectories of IBSMC-PHD; (**b**) Trajectories of CISMC-PHD; (**c**) Trajectories of F-CISMC-PHD.

Then, we conducted 500 Monte Carlo runs for multi-target tracking with the IBSMC-PHD, our CISMC-PHD, and F-CISMC-PHD approaches. The estimated trajectories of all three approaches are demonstrated in Figure 3. We can observe that most of estimated points are covered with the true trajectories in Figure 3b,c, while most of points are not covered with the ground-truth trajectories.

**Figure 4:**
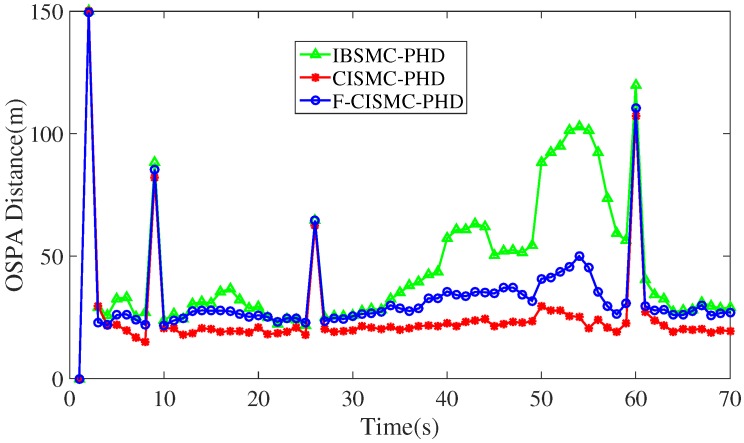
OSPA distances of the IBSMC-PHD, CISMC-PHD and F-CISMC-PHD approaches with clutter number being 10.

Furthermore, Figure 4 depicts the OSPA distances of all three approaches. In these figures, the OSPA distances of the proposed CISMC-PHD and F-CISMC-PHD approaches are smaller than the BSMC-PHD approach. Note that large OSPA distance denotes large tracking error. Thus, the proposed CISMC-PHD and F-CISMC-PHD approaches have the smaller tracking error than the IBSMC-PHD approach. We can also observe that the OSPA distances of the proposed CISMC-PHD and F-CSMC-PHD approaches have four peaks at time 2, 9, 26, and 60. That is to say, targets may appear at these times.

**Figure 5:**
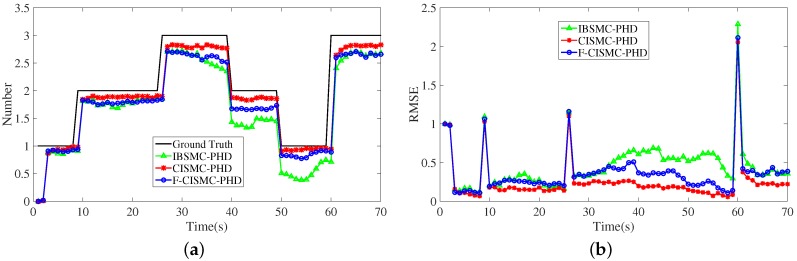
Estimated numbers and RMSEs of IBSMC-PHD, CISMC-PHD and F-CISMC-PHD approaches with clutter number being 10. (**a**) Estimated numbers of the three approaches; (**b**) RMSEs of the three approaches.

We also plot the estimated numbers and corresponding RMSEs of all three approaches in Figure 5. As seen from Figure 5a, the estimated number of the proposed CISMC-PHD approach is closest to the ground truth among three approaches, thus enjoying the lowest RMSE in Figure 5b. In addition, the numerical results of the averaged OSPA distances and RMSEs are listed in Table 5, demonstrating that our CISMC-PHD approach achieves the best estimation on numbers and states. According to Table 5, the F-CIMS-PHD approach can make a compromise between the computational time and estimation accuracy.

### 5.3. Comparison of Estimation Accuracy on Various Numbers of Clutters

**Figure 6:**
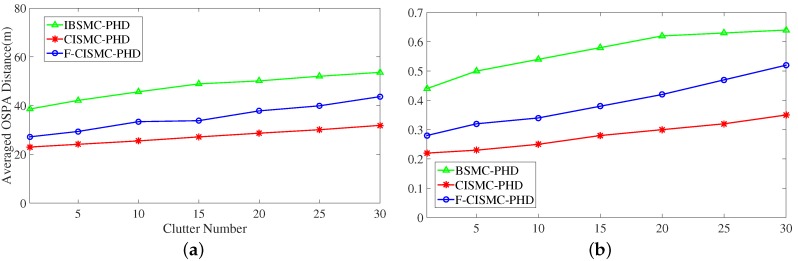
Estimated error of the IBSMC-PHD, CISMC-PHD and F-CISMC-PHD approaches along with the clutter number changing from 1 to 30. (**a**) Averaged OSPA distances; (**b**) Averaged RMSEs of estimated number.

To validate the influence of clutters on multi-target tracking, the IBSMC-PHD, CISMC-PHD and F-CISMC-PHD approaches were implemented with 500 Monte Carlo simulations alongside the clutter number changing from 1 to 30. The results are illustrated in Figure 6a,b. From this figure, we can see that the averaged OSPA distances of all three approaches are enhanced, when the clutter number increases from 1 to 30. Among these approaches, the CISMC-PHD approach has the smallest averaged OSPA distance and RMSE.

### 5.4. Comparison of Estimation Accuracy over Different Probabilities of Detection

In this section, we compare the estimation accuracy at different detection probabilities (varying from 0.92 to 0.98). Here, the clutter number is chosen to be 10, and 500 Monte Carlo simulations are run for the comparison. Table 6 reports the OSPA distances and RMSEs of the IBSMC-PHD, CISMC-PHD and F-CISMC-PHD approaches.

From Table 6, we can observe that the estimated accuracy of the F-CISMC-PHD approach get close to the CISMC-PHD approach, when the probabilities of detection increase from 0.92 to 0.98. Thus, the F-CISMC-PHD approach is suitable for the high probabilities of detection.

### 5.5. Comparison of Estimation Accuracy at Challenging Nonlinear Scenarios

In this section, we compare the estimation accuracy of the IBSMC-PHD, CISMC-PHD and F-CISMC-PHD approaches at challenging nonlinear scenarios. For the challenging nonlinear scenarios, the standard deviation $\sigma_{\theta }$ varies from $\frac{1.5\pi }{180}$ to $\frac{3\pi }{180}$. We implement each approach with 500 Monte Carlo simulations.

The averaged accuracy, evaluated by OSPA and RMSE, is listed in Table 7. Table 7 indicates that the estimation accuracy of all three approaches decreases, when $\sigma_{\theta }$ increases from $\frac{1.5\pi }{180}$ to $\frac{3\pi }{180}$. Compared with the IBSMC-PHD approach, the OSPA distances and RMSEs of the CISMC-PHD and F-CISMC-PHD approaches are smaller at all four values of $\sigma_{\theta }$. It means that the estimation accuracy of the CISMC-PHD and F-CISMC-PHD approaches is more stable than the IBSMC-PHD approach in the challenging nonlinear scenarios.

## 6. Conclusions

In this paper, we have proposed the CISMC-PHD and F-CISMC-PHD approaches, which can estimate the time-varying number and states of multi-target nonlinear tracking. In our CISMC-PHD approach, a novel IS function approximation method is presented, which incorporates a gating method into the CIF method. To initiate the birth intensity of the next recursion, we use the current observations and estimated states to estimate the birth target components. In addition, we also present a fast implementation of the CISMC-PHD approach, namely F-CISMC-PHD, to reduce the time complexity of the CISMC-PHD approach. By clustering the particles into several groups, the target components can be obtained by representing the groups as Gaussian mixture components. Utilizing these components to approximating the IS functions, the computational time can be reduced magnificently. The simulation results demonstrate that the proposed CISMC-PHD and F-CISMC-PHD approaches outperform the conventional BSMC-PHD approach.

This paper concentrates on improving efficiency and accuracy of the conventional SMC-PHD filter. It simply utilizes the multinomial resampling method as the resampling method. Other resampling methods may be integrated in the CISMC-PHD approach for the future work. Besides, the F-CISMC-PHD approach is only suitable for the high probability of detection and small number of clutters. Study on improving the tracking performance of the F-CISMC-PHD approach at low probability of detection and large number of clutters may be seen as another direction of the future work.

## Figures and Tables

**Table 1:** Removing observations generated by the estimated targets.

– **Input:** Observation set $Z_{k}$, and estimated state set $X_{k}^{e}$ – **Output:** Observation set of birth targets ${\tilde{Z}}_{k}$ - **For:** $j=1,2,\cdots ,|X_{k}^{e}|$, 1 Compute the distance $d_{i,j}$ between $x_{k,i}^{e}$ ($x_{k,i}^{e}\in X_{k}^{e}$) and $z_{k,j}$ for each $z_{k,i}\in Z_{k}$ by Equation (37). 2 Extract $z_{k,i}$ satisfying $i=\{i|d_{i,j}<=3\cdot \sigma_{r}\}$. 3 Remove $z_{k,i}$ from $Z_{k}$ to obtain ${\tilde{Z}}_{k}$. - **End For** - Return ${\tilde{Z}}_{k}$.

**Table 2:** The CISMC-PHD filter at time *k*.

– **Input:** Birth particle set ${\left\{x_{k-1,b}^{\left(i\right)},P_{k-1,b}^{\left(i\right)},w_{k-1,b}^{\left(i\right)}\right\}}_{i=1}^{J_{k-1}}$, survival particle set ${\left\{x_{k-1,s}^{\left(i\right)},P_{k-1,s}^{\left(i\right)},w_{k-1,s}^{\left(i\right)}\right\}}_{i=1}^{L_{k-1}}$, and current observation set $Z_{k}$ – **Output:** Target number $M_{k}$, and estimated state set $X_{k}^{e}$ 1 Calculate the IS function $q(x_{k}^{\left(i\right)}|x_{k-1,s}^{\left(i\right)},Z_{1:k})$ of Equation (10) by Equations (33) and (34), and draw particles ${\left\{x_{k|k}^{\left(i\right)},w_{k|k-1}^{\left(i\right)}\right\}}_{i=1}^{L_{k-1}}$ for survival targets by Equations (3) and (4), where $m_{k-1,s}^{\left(i\right)}=\;x_{k-1,s}^{\left(i\right)}$. 2 Approximate the IS function $q(x_{k,b}^{\left(i\right)}|x_{k-1,b}^{\left(i\right)},Z_{1:k})$ of Equation (10) using Equations (33) and (34), and draw particle set ${\left\{x_{k|k}^{\left(i\right)},w_{k|k-1}^{\left(i\right)}\right\}}_{i=L_{k-1}+1}^{L_{k-1}+J_{k-1}}$ for birth particles by Equations (3) and (4), where $m_{k-1,b}^{\left(i\right)}=x_{k-1,b}^{\left(i\right)}$. 3 Calculate $w_{k|k}^{\left(i\right)}$ by Equation (6) for resampling and $w_{k|k}^{\left(i,j\right)}$ by Equation (42) for estimating, using the particle set ${\left\{x_{k|k}^{\left(i\right)},w_{k|k-1}^{\left(i\right)}\right\}}_{i=1}^{L_{k-1}+J_{k-1}}$. 4 Compute $W^{j}$ by Equation (43) with $w_{k|k}^{\left(i,j\right)}$ calculated by step 3 and assign particles into the corresponding group by $W_{th}$ to estimate the target states and number. 5 Estimate the state set $X_{k}^{e}$ by Equation (44) with $w_{k|k}^{\left(i,j\right)}$ and $x_{k|k,j}^{\left(i\right)}$, where $w_{k|k}^{\left(i,j\right)}$ and $x_{k|k,j}^{\left(i\right)}$ belong to the j-th group of step 4. In addition, target number can be approximated by $M_{k}=|X_{k}^{e}|$. 6 Resample particles ${\left\{x_{k|k}^{\left(i\right)},P_{k|k}^{\left(i\right)},w_{k|k}^{\left(i\right)}\right\}}_{i=1}^{L_{k-1}+J_{k-1}}$ to obtain ${\left\{x_{k,s}^{\left(i\right)},P_{k,s}^{\left(i\right)},w_{k,s}^{\left(i\right)}\right\}}_{i=1}^{L_{k}}$ for the next recursion, where $L_{k}=\left[\sum_{i=1}^{L_{k-1}+J_{k}}w_{k|k}^{\left(i\right)}\right]$, and [·] denotes the nearest integer. 7 Remove the observations of survival targets using Table 1, and estimate the birth components by Equations (39) and (40) to obtain the birth component set $B_{k}$. 8 Draw particle from Equation (11) to obtain the birth particles ${\left\{x_{k,b}^{\left(i\right)},P_{k,b}^{\left(i\right)},w_{k,b}^{\left(i\right)}\right\}}_{i=1}^{J_{k}}$ when $B_{k}$ is given. 9 Return $M_{k}$ and $X_{k}^{e}$.

**Table 3:** The construction of target components.

– **Input:** The group weight $W^{j}$, particle $\left\{x_{k,j}^{\left(i\right)},w_{k}^{i,j}\right\}$, ,$j=0,1,\ldots ,|{\hat{Z}}_{k}|$, and $i=1,2,\ldots ,L_{k-1}+J_{k}$ – **Output:** The target components ${\left\{G_{k}^{\left(a\right)},m_{k,a},P_{k,p}\right\}}_{a=1}^{N_{s,k}}$. 1 Initiate the weight of the target component $G_{k}^{\left(1\right)}=0$,$N_{s,k}=0$. 2 Normalize $W^{j}$ by ${\tilde{W}}^{j}=\frac{W^{j}}{\max_{j}W^{j}}$ 3 **for:** $j=0,1,\ldots ,|Z_{k}|$ **if** ${\tilde{W}}^{j}>T_{g}$ – Add the number of target components $N_{s,k}=N_{s,k}+1$. – Substitute $\left\{w_{k}^{\left(i,j\right)}\right\}$ and $\left\{x_{k}^{\left(i\right)}\right\}$ into Equation (44) to approximate the mean $x_{k,N_{s,k}}$ and covariance $P_{k,N_{s,k}}$. – Save the weight of the current component as $G_{k}^{\left(N_{s,k}\right)}={\tilde{W}}^{j}$. 4 Return the target components ${\left\{G_{k}^{\left(a\right)},m_{k,a},P_{k,p}\right\}}_{a=1}^{N_{s,k}}$.

**Table 4:** Initial states of the targets.

Target	State	Appearing(s)	Disappearing(s)
1	[320,5,320,5,0]	1	40
2	[400,-5,400,5,0]	8	50
3	[375,5,375,-5,0]	25	70
4	[400,5,325,-5,0]	59	70
5	[325,-5,375,5,0]	59	70

**Table 5:** Averaged Estimation Errors and Computational Times per 100 particles.

Approaches	OSPA (m)	RMSE	Time (s)
IBSMC-PHD	46.20	0.44	0.02
CISMC-PHD	25.48	0.24	0.2
F-CISMC-PHD	31.93	0.30	0.06

**Table 6:** Tracking Performance over different detection probabilities.

	$p_{d}=0.92$			$p_{d}=0.94$		
	IBSMC-PHD	CISMC-PHD	F-CISMC-PHD	IBSMC-PHD	CISMC-PHD	F-CISMC-PHD
OSPA(m)	52.13	34.40	40.21	43.55	27.39	29.82
RMSE	0.54	0.37	0.47	0.47	0.32	0.37
	$p_{d}=0.96$			$p_{d}=0.98$		
	**IBSMC-PHD**	**CISMC-PHD**	**F-CISMC-PHD**	**IBSMC-PHD**	**CISMC-PHD**	**F-CISMC-PHD**
OSPA(m)	43.55	27.39	29.82	39.35	23.20	23.02
RMSE	0.42	0.27	0.31	0.35	0.22	0.24

**Table 7:** Tracking Performance over different $\sigma_{\theta }$.

	$\sigma_{\theta }=\frac{1.5\pi }{180}$			$\sigma_{\theta }=\frac{2\pi }{180}$		
	IBSMC-PHD	CISMC-PHD	F-CISMC-PHD	IBSMC-PHD	CISMC-PHD	F-CISMC-PHD
OSPA(m)	49.62	27.32	29.66	50.40	27.64	29.99
RMSE	0.43	0.33	0.37	0.47	0.34	0.38
	$\sigma_{\theta }=\frac{2.5\pi }{180}$			$\sigma_{\theta }=\frac{3\pi }{180}$		
	**IBSMC-PHD**	**CISMC-PHD**	**F-CISMC-PHD**	**IBSMC-PHD**	**CISMC-PHD**	**F-CISMC-PHD**
OSPA(m)	51.04	27.43	30.74	52.56	28.02	31.3
RMSE	0.50	0.35	0.39	0.45	0.35	0.39

## References

[1] Yu H., Meier K., Argyle M., Beard R.W. (2015). Moving-Target Tracking in Single-Channel Wide-Beam SAR. IEEE/ASME Trans. Mech..

[2] Wu J., Hu S., Wang Y. (2003). Adaptive multifeature visual tracking in a probability-hypothesis-density filtering framework. Signal Process..

[3] Uney M., Clark D.E., Julier S.J. (2013). Distributed Fusion of PHD Filters via Exponential Mixture Densities. IEEE J. Sel. Top. Signal Process..

[4] Pulford G. (2005). Taxonomy of multiple target tracking methods. IEE Proc. Radar Sonar Navig..

[5] Yang B., Xu G., Jin J., Zhou Y. (2012). Comparison on EKF and UKF for geomagnetic attitude estimation of LEO satellites. Chin. Space Sci. Technol..

[6] Yang B., He F., Jin J., Xiong H., Xu G. (2014). DOA estimation for attitude determination on communication satellites. Chin. J. Aeronaut..

[7] Blackman S. (2004). Multiple Hypothesis Tracking for Multiple Target Tracking. IEEE Trans. Aerosp. Electron. Syst..

[8] Bar-Shalom Y., Li X.R. (1995). Multitarget-Multisensor Tracking: Principles and Techniques.

[9] Roecker J. (1994). Suboptimal Joint Probabilistic Data Association. IEEE Trans. Aerosp. Electron. Syst..

[10] Bar-Shalom Y., Li X.R., Kirubarajan T. (2001). Estimation with Applications to Tracking and Navigation: Theory Algorithms and Software.

[11] Puranik S., Tugnait J.K. (2007). Tracking of Multiple Maneuvering Targets using Multiscan JPDA and IMM Filtering. IEEE Trans. Aerosp. Electron. Syst..

[12] Musicki D., Scala B.L. (2008). Multi-target Tracking in Clutter without Measurement Assignment. IEEE Trans. Aerosp. Electron. Syst..

[13] Yang J., Ji H., Fan Z. (2013). Probability hypothesis density filter based on strong tracking MIE for multiple maneuvering target tracking. Int. J. Control Autom. Syst..

[14] Mahler R. Engineering statistics for multi-object tracking. Proceedings of the 2001 IEEE Workshop on Multi-Object Tracking.

[15] Mahler R. (2003). Multitarget Bayes filtering via first-order multitarget moments. IEEE Trans. Aerosp. Electron. Syst..

[16] Mahler R. A Survey of PHD Filter and CPHD Filter Implementations. Proceedings of the Signal Processing, Sensor Fusion, and Target Recognition XVI.

[17] Mahler R. (2004). “Statistics 101” for multisensor, multitarget data fusion. IEEE Aerosp. Electron. Syst. Mag..

[18] Vo B.N., Singh S., Doucet A. (2005). Sequential Monte Carlo Methods for Multitarget Filtering with Random Finite Sets. IEEE Trans. Aerosp. Electron. Syst..

[19] Mahler R. (2007). Statistical Multisource-Multitarget Information Fusion.

[20] Ristic B., Clark D., Vo B.-N., Vo B.-T. (2012). Adaptive Target Birth Intensity for PHD and CPHD Filters. IEEE Trans. Aerosp. Electron. Syst..

[21] Ristic B., Clark D., Vo B.N., Vo B.T. Improved SMC implementation of the PHD filter. Proceedings of the 2010 13th Conference on Information Fusion (FUSION).

[22] Candy J.V. (2011). Bayesian Signal Processing: Classical, Modern and Particle Filtering Methods.

[23] Morelande M. A sequential Monte Carlo method for PHD approximation with conditionally linear/Gaussian models. Proceedings of the 2010 13th Conference on Information Fusion (FUSION).

[24] Whiteley N., Sumeetpal S., Godsill S. (2010). Auxiliary Particle Implementation of Probability Hypothesis Density Filter. IEEE Trans. Aerosp. Electron. Syst..

[25] Yoon J.H., Kim D.U., Yoon K.J. (2012). Efficient importance sampling function design for sequential Monte Carlo PHD filter. Signal Process..

[26] Chandra K.P.B., Gu D.W., Postlethwaite I. (2013). Square Root Cubature Information Filter. IEEE Sens. J..

[27] Arasaratnam I., Haykin S. (2009). Cubature Kalman Filters. IEEE Trans. Autom. Control.

[28] Mutambara A.G.O. (1998). Decentralized Estimation and Control for Multi-Sensor Systems.

[29] Bailey T., Upcroft B., Durrant-Whyte H. Validation gating for non-linear non-Gaussian target tracking. Proceedings of the 2006 9th International Conference on Information Fusion.

[30] Li T., Sun S., Sattar T. (2013). High-speed Sigma-gating SMC-PHD filter. Signal Process..

[31] Sibley G., Sukhatme G., Matthies L. (2006). The Iterated Sigma Point Kalman Filter with Applications to Long Range Stereo. Robot. Sci. Syst..

[32] Zhang H., Jing Z., Hu S. (2010). Gaussian mixture CPHD filter with gating technique. Signal Process..

[33] Reuter S., Meissner D., Wilking B., Dietmayer K. Cardinality balanced multi-target multi-Bernoulli filtering using adaptive birth distributions. Proceedings of the 2013 16th International Conference on Information Fusion (FUSION).

[34] Li T., Sun S., Corchado J.M., Siyau M.F. Random finite set-based Bayesian filters using magnitude-adaptive target birth intensity. Proceedings of the 2014 17th International Conference on Information Fusion (FUSION).

[35] Mahler R. (2013). “Statistics 102” for Multisource-Multitarget Detection and Tracking. IEEE J. Sel. Top. Signal Process..

[36] Vo B.N., Ma W.K. (2006). The Gaussian Mixture Probability Hypothesis Density Filter. IEEE Trans. Signal Process..

[37] Mo L., Song X., Zhou Y., Kang S., Bar-Shalom Y. (1998). Unbiased converted measurements for tracking. IEEE Trans. Aerosp. Electron. Syst..

[38] Schikora M., Koch W., Streit R., Cremers D. (2013). A sequential Monte Carlo method for multi-target tracking with the intensity filter. Advances in Intelligent Signal Processing and Data Mining.

[39] Zhao L., Ma P., Su X., Zhang H. A new multi-target state estimation algorithm for PHD particle filter. Proceedings of the 2010 13th Conference on Information Fusion (FUSION).

[40] Li T., Sun S., Bolić M., Corchado J.M. (2016). Algorithm design for parallel implementation of the SMC-PHD filter. Signal Process..

[41] Van der Merwe R., Wan E. Gaussian mixture sigma-point particle filters for sequential probabilistic inference in dynamic state-space models. Proceedings of the 2003 IEEE International Conference on Acoustics, Speech, and Signal Processing, (ICASSP’03).

[42] Schuhmacher D., Vo B.T., Vo B.N. (2008). A Consistent Metric for Performance Evaluation of Multi-Object Filters. IEEE Trans. Signal Process..

[43] Yoon J.H., Kim D.U., Yoon K.J. (2013). Gaussian mixture importance sampling function for unscented SMC-PHD filter. Signal Process..

